# ICON 2022 Supplement

**DOI:** 10.12669/pjms.38.ICON-2022.5770

**Published:** 2022-01

**Authors:** Farhana Amanullah, Naila Baig-Ansari

**Affiliations:** 1 Farhana Amanullah; 2 Naila Baig-Ansari Guest Editors, ICON 2022 Supplement

COVID-19 pandemic has taken a distinct toll on routine health service provision, taking away precious lives, resources and health staff focus that none of us have ever witnessed before. During these testing times, the Indus Hospital & Health Network quickly adapted itself to ensure our services were available and the demands of COVID-19 treatment, both in the hospital as well as the community, were met. While IHHN rapidly partnered with stakeholders and participated in much needed clinical research including international drug and vaccine trials, the spirit of action and self was exhibited by all our staff, whether frontline, ancillary or support with the same zeal and courage. And thus, it was only befitting that our ICON 2022 theme would be called “Beyond the Call of Duty”, especially dedicated to the spirit of intense courage and personal sacrifice that our health staff displayed throughout the difficult COVID-19 waves.

Our journal supplement also carries this theme, as it highlights work that clinicians and researchers from over 22 specialties have produced above and beyond their routine work in this all-consuming pandemic. We have deliberately opted for only an online version of this supplement as a direct tribute to the global medical community’s swift adaptation to virtual conferences and online meetings as well as a reflection of Indus’ paperless hospital concept. Writing an academic article requires collaboration, expertise and time. It is commendable that those who submitted their articles managed to carry out their clinical duties as well as coordinate their academic work during the pandemic’s multiple waves. Our publication committee received 32 submissions that underwent double blind peer review. Fourteen original articles, three case reports, one correspondence and one review article were accepted for publication. The articles were diverse and not necessarily focused towards COVID-19. In fact, only four articles are related to the pandemic even though a lot more COVID-19 related research took place at IHHN.

We understand that when international collaborations take place or there is unique research from Pakistan, authors prefer submitting to international indexed journals over local ones. This phenomenon will change when more and more Pakistani journals are indexed and the turnover time for publication is reduced. As such, we are especially thankful to all the authors for their faith in submitting an article to this conference supplement to showcase IHHN’s research capabilities. We are also grateful to our publication committee for their detailed reviews and feedback for improving manuscripts and for collaborating with other reviewers. Our excellent publication coordinator ensured efficient correspondence between reviewers and authors, as well as with the Pakistan Journal of Medical Sciences’ publication team, to ensure the production of this special ICON edition.

It is our hope that this supplement will continue to encourage and inspire Indus’ clinicians to work towards and contribute to future editions.

